# Indexing for body surface area when assessing kidney function

**DOI:** 10.1093/ndt/gfag063

**Published:** 2026-03-20

**Authors:** Abdulfataah A A Mohamed, Marco van Londen, Jasper Stevens, Hiddo J L Heerspink, Ron T Gansevoort

**Affiliations:** Department of Clinical Pharmacy and Pharmacology, University Medical Center Groningen, University of Groningen, Groningen, The Netherlands; Department of Nephrology, Groningen Kolff Center for Kidney Research, University Medical Center Groningen, University of Groningen, Groningen, The Netherlands; Department of Nephrology, Groningen Kolff Center for Kidney Research, University Medical Center Groningen, University of Groningen, Groningen, The Netherlands; Department of Clinical Pharmacy and Pharmacology, University Medical Center Groningen, University of Groningen, Groningen, The Netherlands; Department of Clinical Pharmacy and Pharmacology, University Medical Center Groningen, University of Groningen, Groningen, The Netherlands; Department of Nephrology, Groningen Kolff Center for Kidney Research, University Medical Center Groningen, University of Groningen, Groningen, The Netherlands

**Keywords:** BSA, drug dosing, GFR, indexed, non-indexed

## Abstract

The glomerular filtration rate (GFR) indexed to a standardized body surface area (BSA) of 1.73 m² is the cornerstone metric for kidney function assessment, allowing comparisons across individuals with diverse body sizes. This review outlines the historical development of BSA estimation methods and the rationale behind indexing GFR. While BSA-indexed GFR is useful for population-level comparisons and chronic kidney disease staging, non-indexed GFR reflects renal clearance and should be preferred for specific purposes, such as drug dosing. In addition, it is discussed whether to use indexed or non-indexed GFR when deciding to accept a kidney for transplantation for a patient with kidney failure, or when assessing GFR over time in individuals with severe weight change. Alternative indexing methods based on lean body mass, total body water, or extracellular fluid volume show theoretical potential but lack established clinical benefit. Future research may explore novel indexing methods that balance physiological accuracy and clinical practicality. For now, BSA-indexed GFR remains the clinical standard to assess and monitor kidney function.

## INTRODUCTION

Measurement of the glomerular filtration rate (GFR) is a fundamental tool for assessing kidney function. To facilitate consistent evaluation and enable comparisons between individuals of varying body sizes, GFR is typically indexed to a standardized body surface area (BSA) of 1.73 m^2^ [1]. Such standardization is not unique for nephrology, since BSA has long served as a scaling metric for several other physiological measures in other areas of medicine such as cardiology, pulmonology, and burn care [2–4]. For example, BSA is used to normalize cardiac output, left ventricular mass, and oxygen consumption, and also for burn victims where the extent of injury is expressed as a percentage of total BSA. Despite the long-standing use, the various methods of calculating BSA and why GFR should be indexed remain topics of discussion [5–7]. In this review, we describe the rationale, the historical development, and clinical application of BSA-indexed GFR. We explore the strengths and limitations of BSA-based approaches and consider whether alternative metrics could be of use in the assessment of kidney function across diverse patient populations.

## HISTORICAL DEVELOPMENT OF BSA AND GFR INDEXING

**Figure 1:**
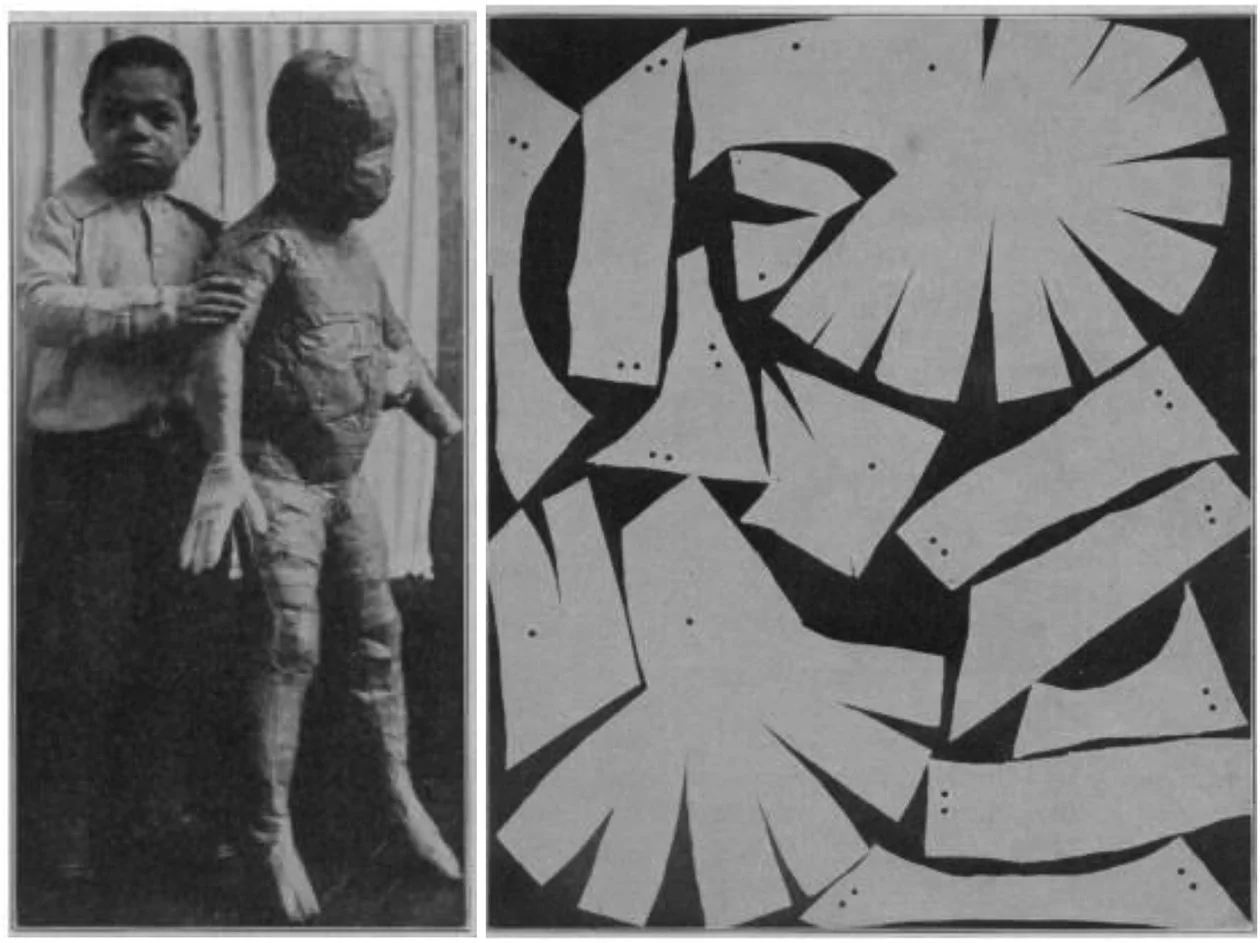
Photographs of the original research of Du Bois and Du Bois showing one of the subjects included in their study to develop a BSA estimation equation [11]. This subject holds the mold of their own surface area (left) and the patterns from the molds that were cut out and weighed for the BSA assessment (right). From DuBois D, DuBois EF. FIFTH PAPER THE MEASUREMENT OF THE SURFACE AREA OF MAN. Arch Intern Med. 1915; XV(5_2):868–881. Public domain.

In 1879, Karl Meeh first published an equation to estimate BSA based primarily on body weight and geometric measurements [8]. This work was conducted as part of his physiological studies investigating the regulation of body heat, where heat loss was directly related to the surface area of the body. Meeh recognized the need for accurate surface area assessment to generalize findings regarding skin functions such as sweat secretion and temperature regulation across subjects of different ages and body types. The result was a practical formula that estimated BSA from body weight alone [8]. Afterward, Eugene Du Bois, physiologist, and his cousin, the engineer Delafield Du Bois, developed in 1916 an equation to better estimate BSA, which would later become one of the most widely used BSA estimation equations [9, 10]. In their study, nine subjects, including one child and one obese subject, were enveloped in paper molds. These molds were then cut open and laid flat on photographic paper exposed to sunlight (Fig. 1). The unexposed sections were removed, and the BSA was calculated based on the weight and the known area density of the photographic paper [11]. The resulting equation provided a simple method to estimate BSA using two readily available anthropometric parameters: height and weight. Owing to its simplicity and practical applicability, it has been used widely in clinical practice for many years.

The practice of indexing kidney function to a fixed BSA of 1.73 m² was first proposed by McIntosh *et al*. in 1928. They examined urea clearance and urine output in individuals of varying height and weight [1]. Their reference BSA value of 1.73 m^2^ was derived using the Du Bois and Du Bois equation and the height and weight of 25-year-old North American men and women from insurance records collected from 1885 to 1912 [9, 12]. Interestingly, recalculations of the reported data suggest the present universal standard of 1.73 m^2^ was a slight miscalculation and should instead have been 1.72 m^2^ [13]. More importantly, recent anthropometric data indicate that the average adult BSA (for the age range 20–29 years) has increased due to increases in length and weight, attributed to improvements in nutrition, reductions in childhood illness, and increases in obesity, with estimates in the USA in 2025 now closer to 1.92 m² (2.03 m^2^ for men and 1.81 m^2^ for women) [14]. Despite changes in actual BSA over time, the continued use of the 1.73 m² standard remains widely accepted, as the absolute reference value is less critical than maintaining uniformity across studies and clinical practice. Changing the established standard would also pose significant challenges for the comparability of historical and current data, potentially disrupting long-standing diagnostic and treatment frameworks [15].

After the development of the Du Bois and Du Bois equation, over 40 other equations have been developed to improve or simplify BSA estimation, many of them based on data of more subjects [16]. Commonly used equations are summarized in Table 1. The Boyd equation was derived in 1935 from a dataset that included 1114 individuals with a wider range in body sizes [17]. This equation was later refined by Gehan and George in 1970, who used data of 401 subjects from Boyd’s research. They observed that the Du Bois and Du Bois equation tends to overestimate BSA in larger individuals, whereas the Haycock equation, developed in 1978 from 81 subjects ranging from premature infants to adults of diverse ethnicities, suggests that Du Bois underestimates BSA in individuals with small body sizes. The Mosteller equation, introduced in 1987 as a simplified calculation based on the Gehan and George equation, was designed for ease of use with pocket calculators [18]. Additionally, population-specific equations have been developed, such as the Yu equation, developed in 270 Taiwanese adults using 3D scanners, and the Hu equation developed in 100 Chinese adults with the paper cast method. For pediatric patients, the Haycock equation is frequently used, which is derived from 81 children of various ages and physiques, including thin to obese individuals.

**Table 1:** Overview of commonly used BSA equation.

Formula	Year	Equation	N	Demographics	Key notes
Meeh [8]	1879	BSA = 0.123123 × weight^2/3^	16	16 males (aged 6 days to 66 years)	First equation used to estimate BSA. Weight-only equation.
Du Bois [9]	1916	BSA = 0.007184 × weight^0.425^ × height^0.725^	9	6 males, 3 females (adults, including 1 child and 1 obese)	Basis for most BSA-indexed clinical calculations. Used widely in adult populations.
Boyd [17]	1935	BSA = 0.017827 × weight^0.4838^ × height^0.5^	1114	Large cohort with broad age range (ranging from infants to adults).	Most calculations based on 231 subjects with complete data.
Gehan and George [19]	1970	BSA = 0.02350 × weight^0.51456^ × height^0.42246^	401	229 infants, 42 children and 130 adults	Based on data of 401 individuals from Boyd’s study.
Haycock [20]	1978	BSA = 0.024265 × weight^0.5378^ × height^0.3964^ ×	81	Infants extending all the way to adults	Derived and validated on subjects from premature infants to adults; shows improved accuracy in smaller body sizes compared to Du Bois equation.
Mosteller [18]	1987	(weight^0.5^ × height^0.5^)/60	-	NA	Simplified modification of Gehan and George equation.
Hu [21]	1999	BSA = 0.0124 * weight + 0.0061 × height–0.0099	100	50 Males, 50 Females	One of the few linear BSA equations.
Shuter and Aslani [22]	2000	BSA = 0.00949 × Height^0.655^ × Weight^0.441^	42	NA	Calculated by a reanalysis of the full Du Bois and Du Bois dataset (including previously omitted data).
Livingston and Lee [23]	2001	BSA = 0.1173 × weight^0.6466^	47	18 Males, 29 Females	Weight-only equation derived to improve BSA prediction in obese patients.
Yu *et al*.[24]	2010	BSA = 0.00713989 * Height^0.7437^ × weight^0.4040^	270	135 Males, 135 Females	Developed using whole body 3D scanners.

**Table 2:** Overview of most common BSA alternatives.

Name	Advantages	Disadvantages
Lean body mass [25, 26, 27, 28]	Reflection of metabolic load.Estimation possible	Estimating equations are not accurate Specialized equipment necessaryDirect measurement is challenging
Total body water [29, 30]	Composes both intracellular and extracellular waterLess dependent on body surface distribution	Does not reflect different metabolic load of different compartmentsSpecialized equipment necessary
Extracellular fluid volume [31, 32, 33]	Main parameter for drug dosingLess dependent on body surface distribution	Does not reflect metabolic load of intracellular compartmentSpecialized equipment necessary

Importantly, there is no single BSA equation universally superior across all populations and clinical scenarios. Different equations may therefore be more appropriate depending on factors such as age, body size, ethnicity, and clinical context [34]. The selection of a specific BSA estimation method should consider the patient population and purpose of use. Nevertheless, the Du Bois and Du Bois equation, developed in 1916 from only a very limited number of individuals, remains one of the most widely used [9]. Whilst being one of the oldest equations and having been based on only a small number of subjects, it has been proven to be reasonably accurate in estimating BSA. Moreover, it is relatively easy to use, and it allows for comparisons over time [35, 36]. Because of these arguments, the Du Bois and Du Bois equation remains an attractive method for BSA assessment in clinical practice.

## ADVANTAGES OF USING BSA-INDEXED GFR

**Figure 2:**
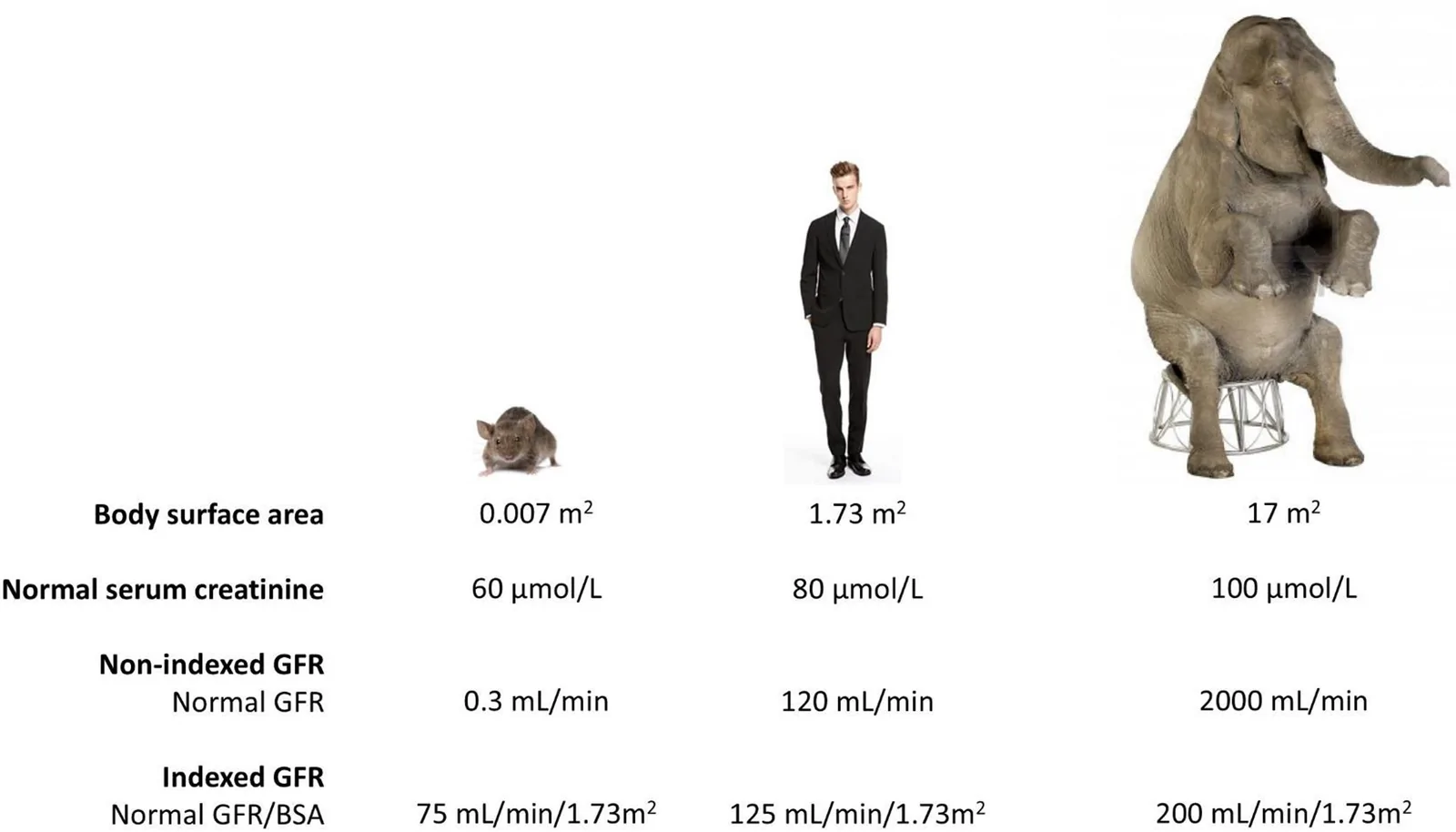
Comparison of non-indexed glomerular filtration rate (GFR) and body surface area (BSA) across species (mouse, human, elephant), illustrating how BSA indexing to a standard of 1.73 m² improves comparability of GFR.

Metabolic rate determines the production of filtrable waste products such as urea and creatinine, necessitating an adequate GFR to maintain homeostasis [37, 38]. GFR closely scales with kidney size, which correlates with body size across individuals and species. This physiological relationship forms the basis for BSA indexing and aligns with the “symmorphosis” principle, where biological design is optimized to match functional needs [39, 40]. Indexing GFR for BSA allows therefore comparison of kidney function between subjects with a different body size [37], whereas non-indexed GFR (absolute GFR expressed in ml/min) is heavily influenced by body size, making direct comparisons between individuals (and even species) complicated. Figure 2 shows the concept of indexing for BSA. When looking across species, a healthy mouse, human, and elephant have serum creatinine values that are remarkably similar [41–43]. However, they have vastly different non-indexed GFRs of approximately 0.3, 120, and 2000 ml/min, respectively. When indexing GFR for the actual BSA of 0.007, 1.92, and 17 m², respectively, and expressing it per standard BSA of 1.73 m^2^, these GFR values become much more comparable with values of 75, 120, and 200 ml/min/1.73 m^2^ [29, 38, 44–48]. Because between humans, differences in body size are far less extreme, the normal BSA-indexed GFR is more or less similar between small, normal, and large humans although their non-indexed GFRs will differ considerably. Because of this principle that indexing for BSA makes kidney function comparable between people of different sizes, the Kidney Disease: Improving Global Outcomes (KDIGO) guidelines recommend the use of GFR values indexed to 1.73 m² for CKD assessment and staging [49, 50].

The advantage of using indexed instead of non-indexed GFR is also illustrated by the clinical case when deciding whether to accept a donor kidney for a transplant candidate. Although kidneys from donors with low BSA have a lower non-indexed GFR than their BSA-indexed GFR suggests, the post-transplant impact for the recipient from such a BSA mismatch is generally smaller than intuitively expected [51, 52]. For example, consider a case of a healthy 30-year-old man with a height of 1.50 m, weight 50 kg, BSA 1.43 m², and an eGFR of 82 ml/min and 100 ml/min/1.73 m². For a single kidney this means 41 ml/min and 50 ml/min/1.73 m². When this man would donate his kidney to a 30-year-old man with height 1.90 m, weight 80 kg, and BSA 2.08 m², this recipient would immediately after transplantation have an eGFR of 41 ml/min, and a relatively low indexed eGFR of 34 ml/min/1.73 m². Because the transplanted kidney, having developed in the context of a smaller body, needs to provide filtration for a much larger metabolic demand, it will gradually adapt. Over time the eGFR of the recipient will therefore approach a value close to 50 ml/min/1.73 m², trying to match the metabolic needs of the recipient as good as possible [51, 53]. In line, a recent systematic review showed that there is no clear association between BSA mismatch and kidney function or graft survival [52]. It should be stated that the quality of included studies was judged to be low. But the study with the best data investigated in 293 living donor transplant procedures in donors as well as recipients not only eGFR but also mGFR and it did so pre-donation as well as 3.5 months and 5 years post-donation [51]. The authors concluded that kidneys of living donors adapt to the recipient’s body size and demands without detrimental effects on kidney function and outcome. These data indicate that, when selecting a kidney donor for a specific recipient, the use of non-BSA indexed GFR should not be emphasized. Instead, long-term kidney function in the recipient is often better predicted by the donor’s standard BSA-indexed eGFR.

## WHEN TO USE BSA-INDEXED OR NON-INDEXED GFR

Despite its utility for standardizing comparisons, BSA-indexed eGFR has limitations in certain contexts, such as when monitoring kidney function over time, especially in populations with changing body sizes, and for drug dosing. Notably, the accuracy of non-indexed eGFR compared to non-indexed measured GFR (mGFR) is similar to that of BSA-indexed eGFR compared to BSA-indexed mGFR, highlighting that indexing itself does not significantly change estimation accuracy [54].

**Figure 3:**
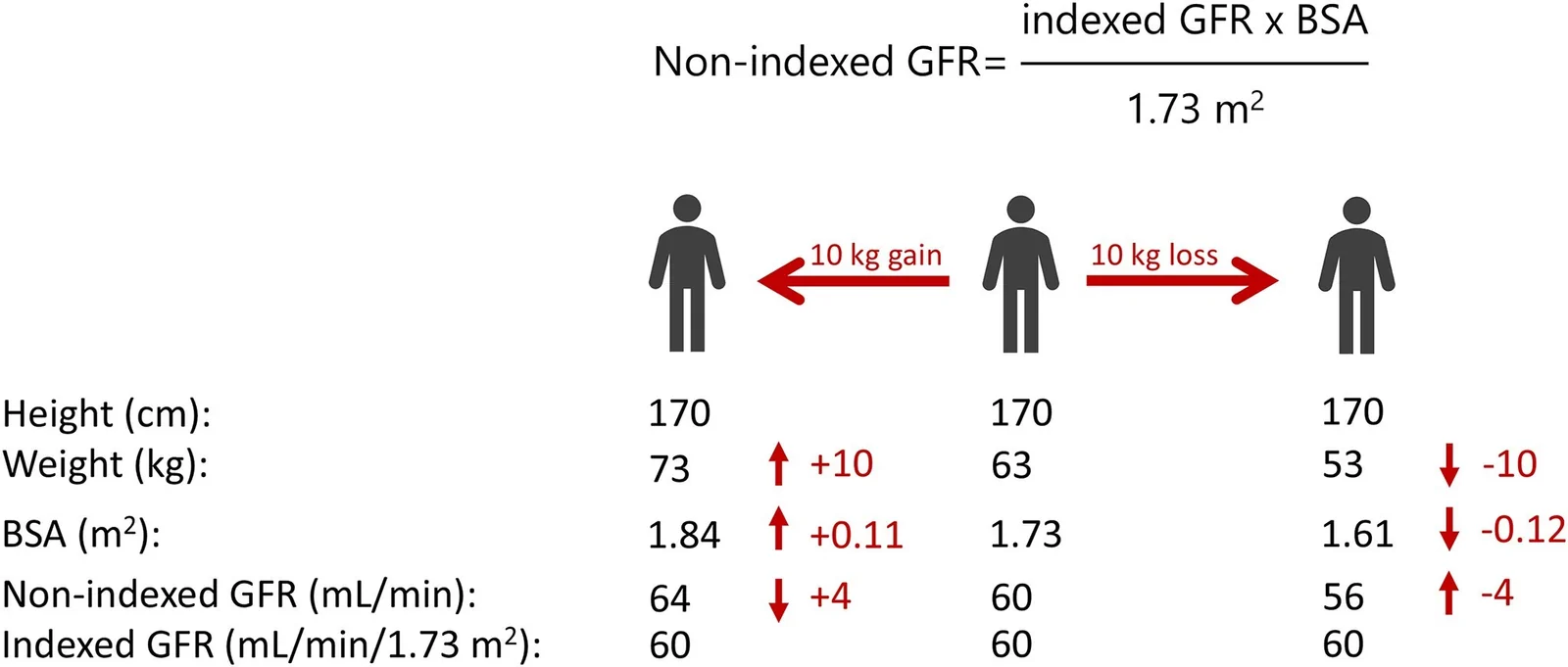
Impact of weight change on body surface area (BSA) and glomerular filtration rate (GFR). The kidneys will adapt proportionally to the change in metabolic demand. Despite differences in non-indexed GFR after weight change, indexing to the standard BSA of 1.73 m² will therefore yield similar GFR values.

In longitudinal follow-up assessing the progression of chronic kidney disease, particularly when using eGFR slope as a surrogate endpoint for kidney function decline, change in BSA should be considered. When height and weight do not change, routinely indexed creatinine or cystatin C based eGFR can be used for comparison of GFR over time, of course with the premise that muscle mass and total cell mass as non-GFR determinants of creatinine and cystatin C, respectively, do not change either. However, in dynamic longitudinal contexts, where fluctuations in BSA due to weight loss may occur, the situation becomes complex. BSA changes, but also kidney function will adapt to weight changes to match metabolic rate. True indexed GFR (as assessed by mGFR) is therefore expected to remain stable while non-indexed GFR changes proportionally to BSA change, reflecting appropriate physiological adaptation (as illustrated by Fig. 3) [55]. The problem arises with eGFR when weight loss is accompanied by disproportionately lower creatinine and cystatin C concentration due to non-GFR determinants (muscle and adipocyte loss), leading to falsely elevated indexed eGFR despite stable true kidney function [56]. This can introduce artifacts that distort the true trajectory of kidney function. For instance, this problem may occur in randomized controlled clinical trials in which an actively treated arm will receive weight loss-inducing agents and in which the control group will receive placebo [57, 58]. Assessing change in kidney function over time in these trials will be challenging because weight loss will likely also result in a loss of muscle mass, which will lower serum creatinine concentration by non-GFR mechanisms. The issue is whether it is proportional to the amount of weight loss and the consequent change in metabolic rate. When it is disproportionately much, it will lead to a falsely high indexed eGFR value. Using cystatin C instead of creatinine to estimate GFR will not offer a solution, because adipocytes produce cystatin C [59]. With weight loss resulting primarily in a loss of adipocytes, this will also result in lower cystatin C values by non-GFR mechanisms and in turn lead to an increase in indexed eGFR. In clinical situations with severe weight loss, or in trials with severe weight loss in one or both treatment groups, it may therefore be better to assess indexed mGFR by using exogenous GFR markers such as inulin, iothalamate, or iohexol instead of estimating GFR with endogenous markers such as creatinine and/or cystatin C. Prospective clinical trials comparing effects of new body weight lowering therapies on changes in eGFR and mGFR will help guide future clinical and research practice.

Another consideration applies for the pediatric populations, where rapid growth leads to an increase in BSA. In these populations non-indexed eGFR and mGFR will increase, whereas BSA-indexed eGFR and mGFR remain stable over time from the age of 2 years onward [60, 61].

Because glomerular filtration is meant to excrete metabolic waste products from the body and because metabolism is related to BSA, indexing mGFR to BSA may be more appropriate than using non-indexed mGFR when assessing changes in kidney function during significant weight loss. In that respect it is important to note that although non-indexed mGFR often declines after major weight loss, the concomitant reduction in BSA and metabolic burden means that indexed mGFR typically remains stable [55, 62]. This correctly reflects preserved filtration capacity relative to the new body size. Consequently, indexed mGFR seems the most appropriate method of assessing longitudinal kidney function changes in case of relevant changes in body weight [62]. When serial mGFR values are not available to assess the renoprotective effect of a treatment in a randomized clinical trial in which significant weight loss occurs in actively treated subjects, it may be of help to analyze BSA indexed as well as non-indexed eGFR values over time [63].

Importantly, GFR indexed for BSA does not accurately represent actual renal clearance capacity for drugs [64–67]. For medicines that are predominantly cleared by the kidneys and have a narrow therapeutic index (e.g. many chemotherapeutic agents and aminoglycosides), steady-state plasma concentrations depend on dose, dosing interval, and absolute drug clearance, but not on volume of distribution or BSA [68]. Therefore, non-indexed GFR (whether estimated or, preferably, measured) provides the most accurate reflection of individual renal elimination capacity and should be used for dose calculation in such cases [66, 69–72]. For instance, in obese individuals, GFR indexed to the standard BSA of 1.73 m² will underestimate true kidney function due to their high actual BSA, which inflates the denominator of the indexing equation [7, 37, 73, 74]. In addition the CKD-EPI formula underestimates the mGFR at severely increased BMI (>40 kg/m^2^) [75]. Although BMI is not necessarily the most accurate measure to define extreme body size in lieu of BSA thresholds, there should be caution for severely obese individuals (BMI >40 kg/m^2^) as they are at risk of underdosing with renally excreted medication, whereas severely underweight individuals (BMI <16 kg/m^2^) are at risk to receive too high doses of such medication. An additional practical consideration when calculating BSA, particularly in relation to GFR indexation and BSA-based drug dosing, is which body weight should be used: actual body weight or adjusted body weight. The latter concept has been introduced because in patients with obesity, actual bodyweight overestimates the metabolically active part of the body (adipose tissue is less metabolically active). The adjusted body weight lies between ideal body weight and actual body weight. It is estimated as IBW + 0.4 × (ABW—IBW), where IBW stands for ideal bodyweight and ABW for actual bodyweight. Although the use of adjusted bodyweight is conceptually attractive, in most clinical and research settings, BSA is calculated using ABW as there is a lack of proven advantage of adjusted body weight over ABW. This approach is also recommended by the American Society of Clinical Oncology (ASCO) for calculating BSA in chemotherapy dosing, as use of ABW in obese patients has not shown increased toxicity, and capping the dose at a BSA of 2 m² has not been demonstrated beneficial [76, 77].

**Figure 4:**
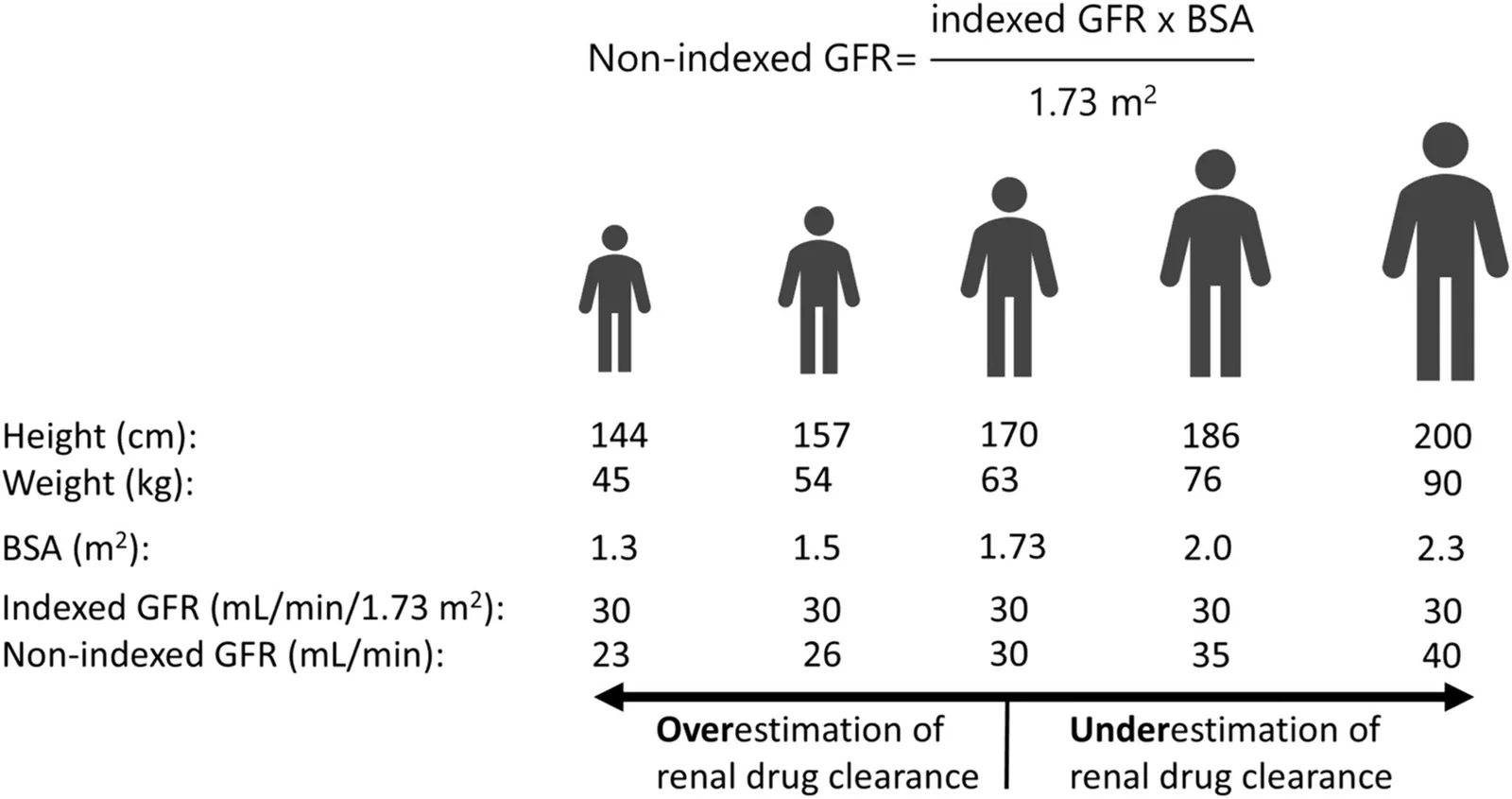
Illustration of the effect of differences in body surface area (BSA) for dosing of drugs that are cleared by the kidneys and that have a small therapeutic window. Similar indexed GFR, but different GFR after de-indexing reflecting differences in absolute renal clearance. For drug dosing, focusing on indexed GFR will therefore lead to overestimation of actual renal excretory capacity in small subjects, and underestimation in large subjects, with potential negative consequences for drug dosing. These effects become clinically relevant especially in patients with impaired kidney function who are very small or very large.

To obtain non-indexed GFR for drug-dosing the most used option in clinical practice is to back-calculate creatinine-based estimated GFR to a non-indexed GFR value (non-indexed GFR = indexed GFR * BSA/1.73). This is needed because the most commonly used GFR estimation equations automatically provide BSA-indexed values (expressed as ml/min/1.73 m^2^)_._ Importantly, clinicians must consistently specify whether GFR is indexed or non-indexed, as using the wrong unit (ml/min vs ml/min/1.73 m^2^) readily leads to misinterpretation. This conversion is only important in individuals at the extremes of body size such as those who are very small or very large, where BSA deviates substantially from the standard 1.73 m². Figure 4 shows that in patients with normal or near-normal BSA, the difference between indexed and non-indexed GFR is minimal and unlikely to significantly impact clinical decisions [76]. Alternatively, kidney function for drug dosing can be assessed as the urinary creatinine clearance expressed in ml/min. However, this requires the collection of a 24-h urine sample, which can be prone to collection errors [49]. Moreover, it should be considered that creatinine is secreted by transporters in the luminal membrane of cells of the proximal tubule in addition to glomerular filtration [78]. Twenty-four-hour creatinine clearance is therefore the resultant of not only glomerular filtration, but also of tubular clearance of creatinine that may contribute 10 to even 40% of total urinary creatinine clearance [49, 79]. Of note, the proportion that tubular secretion contributes to total creatinine clearance increases with decreasing kidney function. This leads to unpredictable GFR overestimation, particularly in severe renal impairment, where accurate GFR assessment is critical for drug dosing. Tubular creatinine secretion can be blocked by using cimetidine or trimethoprim [78]. When using these drugs, 24-h creatinine clearance is a good approximation of true GFR.

Another clinical scenario where a difference between indexed and non-indexed eGFR can lead to confusion is the process of deciding when to start dialysis. It should be kept in mind that in a large, obese male with an eGFR of 8 ml/min/1.73 m², a 24-h creatinine clearance of 25 ml/min may falsely suggest still sufficient kidney function, which for some clinicians is reason to delay the start of dialysis. This reasoning reflects two errors. First, 24-h creatinine clearance is not BSA-indexed, which is necessary to correctly assess the need to start kidney replacement therapy because GFR should match metabolic rate (we refer to our example in Fig. 2). Second, 24-h creatinine clearance includes tubular secretion, overestimating GFR. In obese subjects tubular creatinine secretion is even disproportionately increased, further inflating clearance estimates.

For assessing the dose of renally excreted drugs, the Cockcroft-Gault (CG) equation is still sometimes used to estimate non-indexed kidney function, especially in the summary of product characteristics of older medications. Unlike many newer equations, the CG equation incorporates not only sex, age, and serum creatinine, but also body weight, whereas it does not index for BSA. One should realize that the CG equation does not estimate GFR but was designed to estimate 24-h creatinine clearance. Because of the aforementioned contribution of tubular creatinine secretion, the CG equation will overestimate true GFR, especially in patients with impaired kidney function, where an accurate assessment of GFR is most crucial for drug dosing. Additionally, the equation relies on body weight as a surrogate for muscle mass, which causes inaccuracies especially in patients with obesity where it will lead to a significant overestimation of true GFR. Because of these limitations, the CG equation should not be used to estimate kidney function for drug dosing.

For this indication, the use of an exogenous clearance marker to assess non-indexed mGFR can be considered the gold standard. This can be done by modelling GFR from plasma disappearance curves of iohexol, iothalamate, ^51^Cr-EDTA, or ^99m^Tc-DTPA after an intravenous injection of these agents or by measuring GFR as urinary clearance of these markers during continuous infusion with a concentration at steady state. However, such assessments are not often performed in routine clinical practice due to their time-consuming nature, their relatively high costs, invasiveness, and being less patient friendly [80, 81]. However, it should be considered for dosing of drugs that are renally cleared, especially when they have a small therapeutic window and in subjects where creatinine is not a reliable GFR marker. The latter consideration is of importance when there is an important non-GFR determinant of creatinine concentration i.e. in subjects with, for age and sex, clearly too low muscle mass (e.g. in case of sarcopenia or after limb amputations) or too high muscle mass (e.g. in case of a bodybuilder).

## ALTERNATIVE METHODS FOR INDEXING

**Figure 5:**
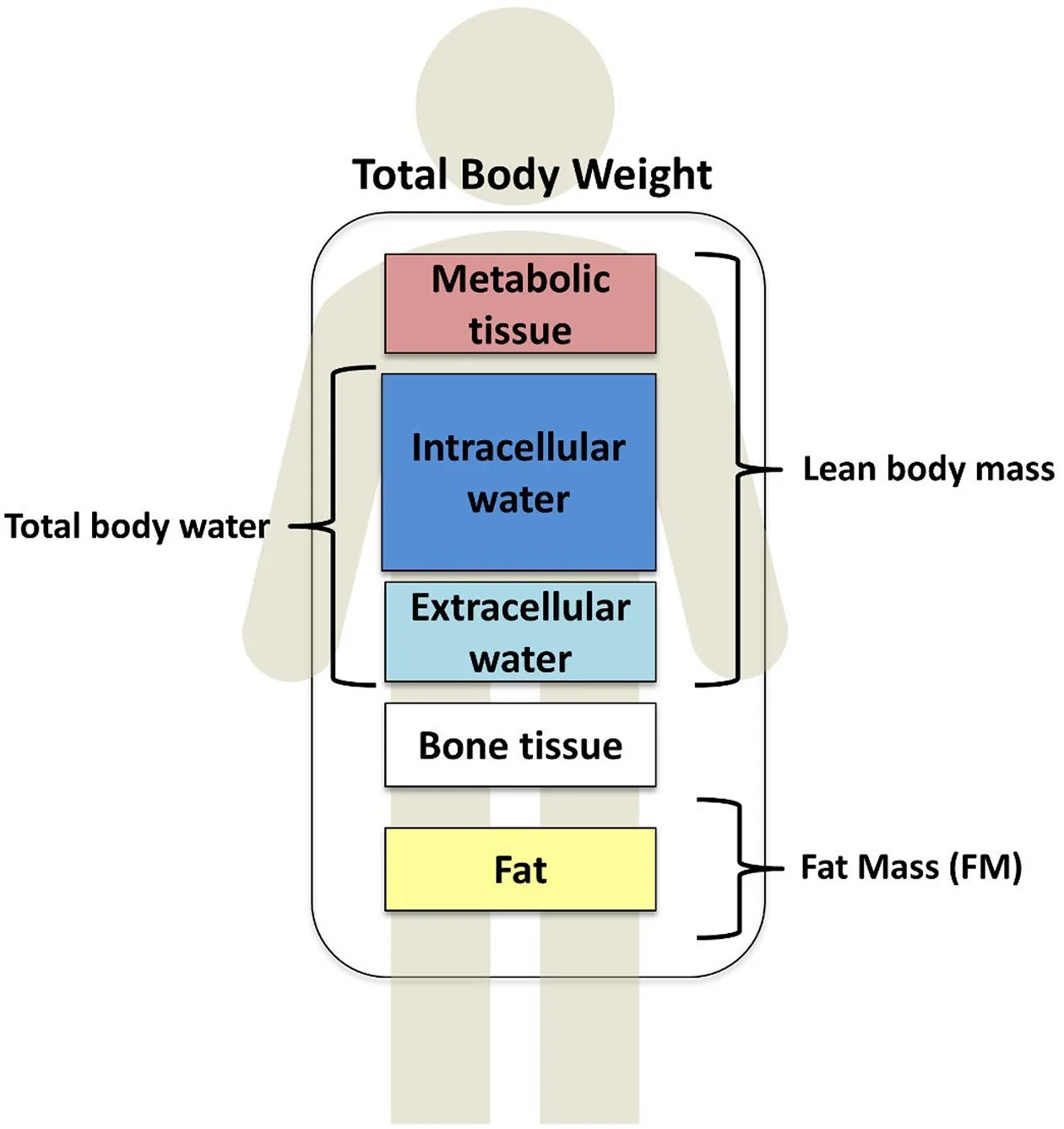
Overview of the body composition.

Alternative methods for indexing have been proposed because they may better reflect the actual metabolic burden placed on the kidneys than BSA, especially in populations with atypical body compositions (Table 2). In this respect lean body mass (LBM), total body water (TBW), and extracellular fluid volume (ECFV) have been investigated [25, 26, 27, 82, 83]. As illustrated in Fig. 5, body composition can be deconstructed into compartments directly relevant for renal workload and filtration capacity. LBM is thought to more closely reflect metabolically active tissue mass, potentially providing a more relevant metric for indexing GFR than BSA, especially in severely obese individuals. Estimation of LBM can be done using simple equations or using bioelectrical impedance analysis (BIA). However, the equations for estimating LBM show discrepancies compared to the gold standard dual-energy X-ray absorptiometry (DEXA) measurements and the results of BIA show large measurement variation between measurement devices, protocols, and individuals [28,84,85] Measurement of LBM can be performed more accurately using DEXA, or alternatively, using whole-body magnetic resonance imaging (MRI), or air-displacement plethysmography [86]. However, these techniques are costly, time-consuming, and, in the case of DEXA, involve ionizing radiation that limits repeated use [84, 87, 88]. Although these measurement methods can provide very precise LBM values when required for research purposes, these methods are impractical for widespread clinical application.

Similarly, TBW and ECFV both have been suggested as measures for indexing GFR [86, 30, 31, 32, 89, 90, 91]. When ECFV is derived from a clearance procedure it should ideally not use the same clearance procedure as the GFR measurement [87]. However, the added value of TBW or ECFV over BSA remains unproven, and their use is as yet not recommended in guidelines for clinical practice. Moreover, despite the theoretical appeal, these alternative indexing methods have not demonstrated clear advantages over the more practical BSA-based indexing in routine clinical practice. Any potentially superior metric would need to offer substantial benefits to justify the challenge of re-standardizing historical and ongoing eGFR and mGFR data across populations and databases. Thus, while promising in specific research contexts, alternative methods remain largely impractical and unproven as superior to BSA for standard GFR indexing.

## FUTURE PERSPECTIVES

Advances in techniques for anthropometric measurements offer new opportunities for refining BSA estimates and enhancing the precision of kidney function assessments [16, 33, 92, 93, 94, 95]. However, these measurement techniques, such as 3D body scanning, whole-body CT scanning, all have the downsides of limited feasibility and high costs [16, 95]. BIA has been proposed as a less costly and non-invasive measurement method of body composition that may aid in the proper indexation of GFR. It is also increasingly used in dialysis patients to evaluate volume status, in gyms to evaluate LBM and muscle mass and by dieticians to evaluate nutritional needs [96]. However, as stated above, the results of BIA analysis have at present limited clinical reliability [96]. Future research should therefore be directed at methodology standardization and minimizing measurement error to allow more widespread clinical use.

Importantly, despite theoretical benefits, alternative GFR indexing methods based on LBM, TBW, or ECV have not proven superior to traditional BSA-indexing in everyday clinical use. Future research should therefore focus on validating alternative methodologies, such as refined estimates of lean tissue mass or fluid compartments using alternative techniques (e.g. DEXA or MRI), and incorporation of direct or proxy measures of metabolic rate, by assessing their feasibility in routine clinical settings, and determining their impact on clinical outcomes. Ideally, GFR would be indexed to an individual’s metabolic rate, as this more accurately reflects the functional demands placed on the kidneys [38]. However, metabolic rate is difficult to measure directly and estimation equations have not been validated [29].

## CONCLUSIONS

At first glance indexing of GFR for BSA may seem curious for many clinicians. However, it has deep physiological meaning. BSA-indexed GFR allows comparing kidney function across individuals with different body sizes in clinical practice. Its physiological basis and widespread acceptance have made BSA-indexed GFR the cornerstone of defining and classifying chronic kidney disease and when performing epidemiological studies. Limitations do exist, particularly regarding drug dosing and possibly the use in populations with considerable changes in body weight. Emerging technologies such as 3D body scanning and BIA present opportunities for more personalized and precise GFR indexing. Nonetheless, their practical implementation is currently limited by cost, accessibility, and measurement imprecision. While future research may explore novel methods for indexing GFR that balance physiological relevance with clinical feasibility to improve kidney function assessment without compromising routine care, the present BSA-indexed GFR estimation equations remain the standard to assess kidney function in clinical care.

## Data Availability

No new data were generated or analyzed in support of this research.
